# End-to-End Residual Network for Light Field Reconstruction on Raw Images and View Image Stacks

**DOI:** 10.3390/s22093540

**Published:** 2022-05-06

**Authors:** Ahmed Salem, Hatem Ibrahem, Bilel Yagoub, Hyun-Soo Kang

**Affiliations:** 1 School of Information and Communication Engineering, College of Electrical and Computer Engineering, Chungbuk National University, Cheongju 28644, Korea; ahmeddiefy@chungbuk.ac.kr (A.S.); hatem@chungbuk.ac.kr (H.I.); bilel.yagoub@cbnu.ac.kr (B.Y.); 2 Electrical Engineering Department, Faculty of Engineering, Assiut University, Assiut 71515, Egypt

**Keywords:** light field reconstruction, based view synthesis, micro-lens image, convolutional neural network

## Abstract

Light field (LF) technology has become a focus of great interest (due to its use in many applications), especially since the introduction of the consumer LF camera, which facilitated the acquisition of dense LF images. Obtaining densely sampled LF images is costly due to the trade-off between spatial and angular resolutions. Accordingly, in this research, we suggest a learning-based solution to this challenging problem, reconstructing dense, high-quality LF images. Instead of training our model with several images of the same scene, we used raw LF images (lenslet images). The raw LF format enables the encoding of several images of the same scene into one image. Consequently, it helps the network to understand and simulate the relationship between different images, resulting in higher quality images. We divided our model into two successive modules: LFR and LF augmentation (LFA). Each module is represented using a convolutional neural network-based residual network (CNN). We trained our network to lessen the absolute error between the novel and reference views. Experimental findings on real-world datasets show that our suggested method has excellent performance and superiority over state-of-the-art approaches.

## 1. Introduction

In comparison to conventional photography, light field (LF) photography is characterized by its capacity to convey additional information about three-dimensional (3D) space. On the other hand, traditional photography records only the 2D projection of visible light by integrating light rays. In contrast, LF captures arrays of light arriving simultaneously from all directions [1,2]. With the introduction of commercial light field cameras and the proliferation of applications such as light field stitching [3], object segmentation [4], de-occlusion [5], post-capture refocusing [6], depth-sensing [7], saliency detection [8], and so on, LF photography has received a tremendous amount of attention.

Prior to the development of commercial LF cameras, LF was recorded using camera arrays [9] and computer-controlled gantries [2]. Commercial LF cameras have provided mobile and cost-effective choices to the issues above through the encoding of angular information of incoming rays through a microlens array upstream of the picture sensor [10,11]. It is impossible to sample LF densely in both the spatial and angular dimensions because of the low resolution of modern LF cameras.

There have been investigations to overcome the resolution constraint and obtain high-resolution (HR) densely sampled LF images. By reconstructing high-resolution LF images from their lower-resolution counterparts (spatial super-resolution) [12,13,14], others have been conducted to reconstruct dense LF images from a limited number of views (angular super-resolution) [15,16,17]. Spatial super-resolution of LF images is outside the scope of our study since we are primarily concerned with LF reconstruction (angular super-resolution). The objective of our approach differs from the NeRF’s view concerning the viewpoint. Our approach aims at reconstructing scenes from fixed viewpoints different from NeRF’s method, which can reconstruct free-viewpoint images [18].

Some studies estimate the depth and subsequently warp input views to reconstruct dense LF images [15,19], whereas others do not explicitly estimate the depth [16,17,20]. On the other hand, depth measurement and warping are complicated techniques, particularly for LF photos with small discrepancies, making it simple to introduce defects and lose image consistency. Other learning-based techniques try to upsample epipolar plane images (EPIs) in many orientations. However, this approach does not completely evaluate angular information and so does not restore texture features. Gul and Gunturk [21] suggested using a lightweight model to double the spatial and angular resolution in LF pictures. Salem et al. [20] proposed a network to improve the angular resolution; however, their network could only reconstruct 7 × 7 views out of 3 × 3 views.

**Figure 1:**
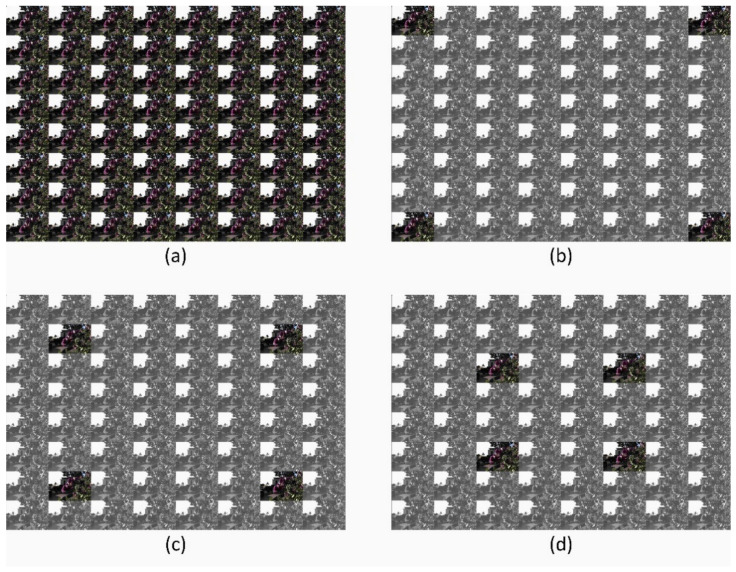
LF input–output relationship for different reconstruction tasks; 8 × 8 views are reconstructed from 2 × 2 views to generate 60 novel views. Colored images represent ground-truth and input views, while gray images represent output views to be reconstructed; (**a**) 8 × 8 ground-truth views are used to train the network for different reconstruction tasks, (**b**) task 1: 2 × 2 − 8 × 8 extrapolation 0, (**c**) task 2: 2 × 2 − 8 × 8 extrapolation 1, (**d**) task 3: 2 × 2 − 8 × 8 extrapolation 2.

Densely sampled LF images are reconstructed from a small number of input views, and the positions of these views are important for distinguishing between different reconstruction (interpolation and extrapolation) tasks. In our research, we propose reconstructing 8 × 8 views from 2 × 2 views with three different orientations depending on the positions of the input views, as shown in Figure 1.

This research presents a deep residual network for densely sampled LF image reconstruction from a limited number of input views to overcome the challenges above and improve the quality of LF reconstruction. We trained the proposed model on raw LF images. The raw LF image is formed by compressing a 2D array of gathered images of the same scene into a single image. The network develops the ability to comprehend and model the connection between several images of the same scene while training on raw images, which leads to better results. Our model is divided into two sequential modules: LF reconstruction (LFR) and LF augmentation (LFA). Each module is modeled using a residual network based on a convolutional neural network (CNN). The LFR module is responsible for reconstructing an initial densely sampled LF. To rebuild high angular resolution LF images, the LFA module investigates the spatial–angular relationships among the initially estimated images.

Numerous experiments demonstrated our model’s advantage in reconstructing high-quality LF images. Our paper makes the following significant contributions:

- We present a deep residual convolutional neural network (CNN) for reconstructing high-quality LF pictures. Our network was built so that it can be used to model different interpolation and extrapolation tasks for LF reconstruction with the same network architecture.
- We fully trained our model using raw LF photos, enabling the network to represent the non-local characteristics of the 4D LF images more effectively. Furthermore, utilizing raw LF pictures simplifies our work by converting it from an image reconstruction to an image-to-image translation.
- Comprehensive experiments on challenging datasets demonstrate our model’s ability to outperform the state-of-the-art methods to reconstruct LF images in different tasks.

## 2. Related Work

LF could be collected by inserting a microlens array in front of the image sensor to encode the angular information included in the incoming rays. Because of the low sensor resolution of LF cameras, it is impossible to obtain densely sampled LF in both the spatial and angular dimensions. Numerous studies have investigated LF angular reconstruction to resolve the inherent compromise between spatial and angular resolution. It can be divided into two groups based on how much the model relies on the scene’s depth.

### 2.1. Depth-Dependent LF Reconstruction

The learning process is split into two steps by depth-dependent methods: depth estimation and LF enhancing. The initial stage is to predict a depth map for each reconstructed image, and this map is then utilized to reconstruct novel views through a warping operation. The reconstructed images are optimized in the second step since the estimated depth is often erroneous and noisy. To limit reconstruction errors, these two steps are trained end-to-end. Numerous conventional ways have been presented to implement this strategy. For example, Wanner and Goldluecke provided a variational framework [21]. They used EPI analysis to estimate depth maps locally in their framework. Then, using convex optimization methods, these depth maps were refined and utilized to rebuild LF pictures. Later on, they refined this approach by recasting the issue as a continuous inverse problem, allowing the inclusion of foreshortening effects [22]. Using a patch-based method, Mitra and Veerara-Ghavan established a common framework for various LF tasks in which they characterized LF patches using a Gaussian mixture model (GMM) [23]. Pendu et al. [24] suggested obtaining depth layers for scene representation using a regularized least squares regression. These layers may be manipulated and filtered to reconstruct images from various viewpoints.

Kalantari et al. [15] simulated the same process through two successive modules to calculate disparity and then inferred colored novel views, one of the learning-based ways of reconstructing a highly sampled LF. To train these networks to reconstruct high-quality pictures from any angle, end-to-end training was utilized. Since this technique reconstructs each view separately, it cannot link different views or give a high-quality reconstruction in blocked regions. Salem et al. [19] further recommended employing a predefined convolutional network at the initial step to save preprocessing time and dual disparity vectors to reduce interpolation error when warping input views to reconstruct output views. This method concentrates on small baseline images, different from Jin et al. [25], who suggested employing depth information to rebuild LF pictures with a broad baseline. This approach involves computing a depth map for each picture to be constructed; after warping all the input views with this depth, the warped images are blended to reconstruct the final views. At the blending step, convolutional layers were used to analyze the spatial and angular dimensions alternately to investigate the directional relationships between distinct pictures, similar to the method utilized in [26].

### 2.2. Depth-Independent LF Reconstruction

Methods that do not rely on depth learn depth information to reconstruct densely sampled LF pictures. Transform-assisted view synthesis assumes that a sparse signal’s frequency representation can be computed using just a fraction of samples. To express an LF as a linear combination of k non-zero continuous angular frequency coefficients implies that it is k-sparse. This algorithm looks for frequency values and coefficients to rebuild the deleted samples [27]. This method has been used to improve the quality of the reconstruction and cut down on the number of samples needed. Vagharshakyan et al. [28] recommended studying light field sampling and reconstitution using the shearlet transform. This approach performed well in scenarios with semi-transparent objects.

Given deep learning’s huge success, various learning-based solutions have been proposed. Examples include Yoon et al.’s [29,30] spatial and angular upsampling approach. This method could only reconstruct 3 × 3 from 2 × 2 views. Except for the center image, reconstruction uses only surrounding views in the horizontal or vertical directions. The quality of reconstruction degrades due to the inefficient utilization of angular information. In [31], the authors recreated several images using 2D alternating spatial–angular convolutions. Because this approach ignores inter-view interactions, it makes wrong shadows and ghosting artifacts at the margins of novel views.

Learning-based approaches have been used in the EPI field because EPIs might be a way to reflect consistency. Wu et al. [29] employed a blur kernel to retrieve the low-frequency components to eliminate ghosting artifacts. A deblur kernel was then employed to restore high-frequency components. They trained another CNN to learn fusion scores for upsampled EPIs with varying shearing values [29,30]. They did not utilize enough angle data since they only used EPIs in one direction. Wang et al. [31] integrated two- and three-dimensional CNNs to produce a pseudo-4D CNN using EPI and EPI stacking. They improved the reconstruction quality by utilizing EPI structure-preserving loss [32]. They only employed horizontal or vertical EPI stacks for the reconstruction, wasting angular data. They also upsampled LF, which caused additional mistakes to accumulate in the final reconstruction views. Liu et al. [16] proposed a multi-angular epipolar-based network using horizontal, vertical, and two angular EPI stacks.

There are a few options for techniques that utilize raw LF images. Gul and Gunturk [33] suggested using a shallow neural network to reconstruct LF images collected by a plenoptic camera. This network was trained to enhance spatial and angular resolution. However, this approach is only effective for achieving a magnification factor of two. Finally, the trained model must be run to reconstruct the LF image for each viewpoint. A light network was suggested by Salem et al. [34] in response to the VDSR network design [35] to super resolve LF images by 2× and 4×. They designed their model to run on raw LF images and have provided acceptable results. Very recently, Salem et al. [20] proposed a residual network to reconstruct LF images on raw images, and they managed to provide good results. Additionally, they used small-baseline LF characteristics by initializing the novel views using the closest input view through the nearest view approach. This approach, however, was confined to a single reconstruction task (7 × 7 views from 3 × 3 views).

## 3. Methodology

### 3.1. Problem Formulation

**Figure 2:**
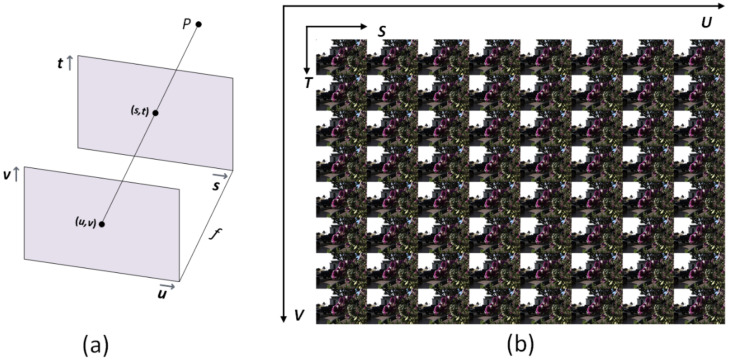
Parameterization and visualization of 4D LF. (**a**) *L*(*u*, *v*, *s*, *t*) denotes the light beam from an arbitrary point P that crosses the two planes at the angular (*u*, *v*) and spatial (*s*, *t*) positions. (**b**) The 4D LF may be seen as a 2D array of view images, with neighboring images differing slightly from one another.

The two-plane parameterization is the most commonly used method for parameterizing the 4D LF [2]; hence, the light ray that crosses the two planes from any point P in the 3D space can be represented as *L*(*u*, *v*, *s*, *t*) where the intersection of the angular and spatial planes are (*u*, *v*) and (*s*, *t*), respectively, as shown in Figure 2. In this research, we aimed to reconstruct densely sampled LF: $L\in \;R^{U\times V\times S\times T}$ from a sparse set of input views $L^{\prime }\in \;R^{u\times v\times S\times T}$. Where the angular resolution of the input LF and reconstructed LF is represented by (*u*, *v*) and (*U*, *V*), respectively. (*S*, *T*) represents the spatial resolution of each input or output view, where *U* > *u* and *V* > *v*. In our method, 8 × 8 views were reconstructed from 2 × 2; hence, views (*u* = *v* = 2, and *U* = *V* = 8).

### 3.2. Raw LF Image and View Image Stack Reconstruction

**Figure 3:**
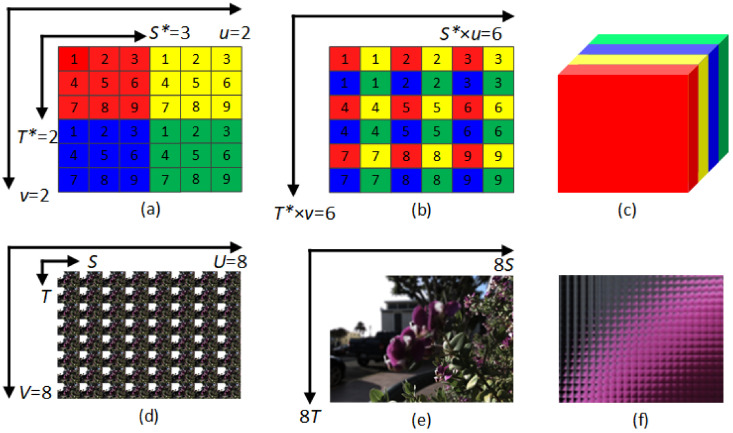
The reconstruction of raw LF image and view image stack; (**a**) 2 × 2 views of LF, where each view is shown using one color and contains 9 pixels from 1 to 9 with size (*u*, *v*, S*, T*) = (2, 2, 3, 3), (**b**) The mapping into a raw LF image of size (*u* × S*, *v* × T*) = (6, 6), (**c**) The mapping into a view image stack of size (*uv*, S*, T*) = (4, 3, 3). (**d**) An example of a 4D LF image captured by a Lytro Illum camera with size (*U*, *V*, *S*, *T*) = (8, 8, 541, 376). (**e**) The raw LF of the image in (**d**) with size (*US*, *VT*) = (8 × 541, 8 × 376). (**f**) A close-up of a portion of the raw LF image shown in (**e**).

We propose generating raw LF images and viewing image stacks to exploit both angular and spatial information adequately. The 2D array of LF pictures is reordered using a periodic shuffling operator (PS) $\in R^{U\times V\times S\times T}$ into the raw LF image $\in R^{US\times VT}$ as proposed in [36]. A similar PS operator is used to rearrange the raw LF image ∈ $R^{US\times VT}$ into a view image stack ∈ $R^{UV\times S\times T}$ as proposed in [37]. An example is given in Figure 3 to explain briefly the 4D representation of LF images in addition to the reconstruction of raw LF images and view image stacks.

### 3.3. Network Architecture

#### 3.3.1. Overview

**Figure 4:**
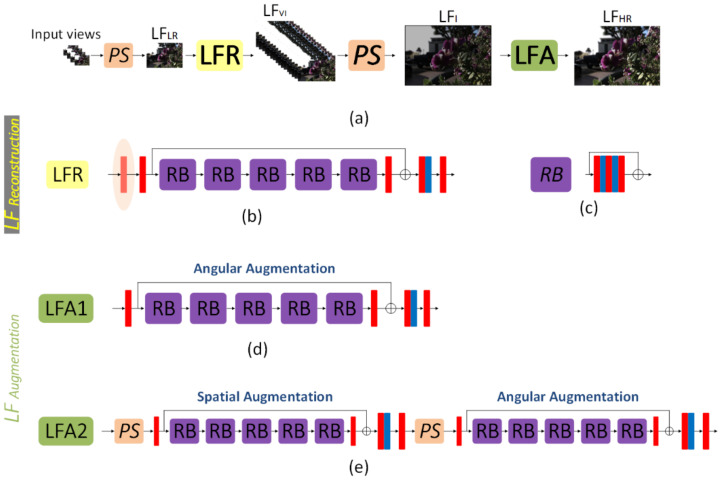
A visual representation of the proposed network architecture. (**a**) The sparse set of input views are rearranged into raw LF representation LF_LR_ using the periodic shuffling operator (PS) to reconstruct the input raw LF image, and this image is then fed into the LF reconstruction block (LFR) to generate the initial LF images LF_VI_. These images are then rearranged into the raw LF representation using the PS. The initial raw LF image LF_I_ is fed into the LF augmentation block (LFA) to improve the quality and generate the final high-resolution image LF_HR_. (**b**) (LFR): this block is responsible for reconstructing dense LF images from a few input images. It can be called a magnifier where the magnification percentage depends on the reconstruction task (number of input images and the number of images to be reconstructed). (**c**) The residual block (RB) is the network’s central unit, consisting of cascaded convolutions and ReLU connected by a skip connection, where the red block represents the convolutional layer (Conv). In contrast, the blue ones represent the rectified linear unit (ReLU). (**d**) (LFA1): this block is responsible for enhancing the quality of the initial reconstructed LF images. It consists of one block that works on the raw LF images to perform an angular augmentation. (**e**) (LFA2): this block performs the same function as the LFA1 block; however, the first main block here works on the view stack images to perform a spatial augmentation rather than working on the raw LF images to perform an angular augmentation. Where LFA1 is used for the extrapolation tasks (task 2: 2 × 2 − 8 × 8 extrapolation 1, and task 3: 2 × 2 − 8 × 8 extrapolation 2.), while LFA2 is used for the interpolation task (task 1: 2 × 2 − 8 × 8 Extrapolation 0) as shown in Figure 1.

The proposed model is divided into two sequential modules: LF reconstruction (LFR) and LF augmentation (LFA), as shown in Figure 4. The LFR module is responsible for reconstructing densely-sampled initial LF images using a sparse set of input views. In contrast, the LFA module is responsible for increasing the quality of the initial images that have been reconstructed. The view stack images are reordered by a periodic shuffling operator (PS) into raw LF images as proposed in [36] or reordering the raw LF image into view stack images, as proposed in [37]. In our method, we reconstruct 8 × 8 views from 2 × 2 views with three different orientations depending on the positions of the input views, as shown in Figure 1.

The 2 × 2 input views are fed to the network as a view image stack with size (4, H, W), where a PS is used to rearrange the input stake to a raw LF image LF_LR_ (low-resolution LF image) with size (2H, 2W). The raw LF image is fed to the LFR to reconstruct 16 raw images with size (16, 2H, 2W) denoted as LF_VI_ (initial LF view image stack). Then, PS rearranges it to the raw LF to obtain the initial LF image LF_I_. The initial LF image LF_I_ is then fed to the LFA to restore texture details and provide better quality for the final output LF image LF_HR_ (high-resolution LF image).

The LFR and LFA networks share the same structure: a convolution layer, multiple residual blocks (RB) with a skip connection (we used five RBs in our implementation for all networks), and another convolution layer. Our RB consists of three convolution layers with two rectified linear units (ReLU) in between, as shown in Figure 4c, which is similar to the RB used in [38], except that their RB consists only of two convolution layers with a ReLU in between. This residual structure with cascaded RBs and a skip connection has been proposed by [39] and is used in a lot of work. With such a structure, it is possible to get rid of low-frequency information in LF_LR_ images through skip connections to the final output. The main parts of the network can instead focus and train on extracting more features and restoring high-frequency components.

We propose two similar designs for the LFA network based on the reconstruction task. LFA1 has one block that works on LF raw images, and LFA2 has two main blocks, one of which works on view image stacks while the other works on LF raw images. As mentioned earlier, LFA1 is employed in the case of extrapolation tasks (tasks 2, 3), while LFA2 is employed in the case of interpolation tasks (task 1). The interpolation task is more challenging since the input LFs are far from the LFs to be reconstructed than the extrapolation task. Accordingly, we applied two augmentation methods for the interpolation task: first, a spatial augmentation was applied on the reconstructed view image stack; second, an angular augmentation was applied on the raw LF image similar to the LFA1.

Specifically, four LF images were fed to our network, where they were rearranged by a PS from a view image stack of size (4, H, W) into a raw LF image LF_LR_ of size (2H, 2W). The LF_LR_ was then fed to the LFR block to generate a view raw image stack LF_VI_ of size (16, 2H, 2W). Using a PS, the LF_VI_ was rearranged into a view image stack of size (64, H, W) representing the initial reconstructed LFs. At this stage, the images corresponding to the input views were replaced after being deformed due to processing in the LFR block with the original input views. Finally, another PS was used to rearrange the view image stack of size (64, H, W) into the initial raw LF image LF_I_ of size (8H, 8W). After that, the LFA block was applied to enhance the quality of the initial reconstructed LFs. In the case of the extrapolation tasks, an angular augmentation was applied only using LFA1, which is a modified version of the residual block (RB) proposed by [39] and the same as the one used in [20], except for the RB, which is bigger in our research. In the case of the interpolation tasks, both spatial and angular augmentations were used. First, spatial augmentation was applied to the view image stack instead of the raw LF image with a cascading structure similar to angular augmentation. The initial reconstructed 64 images represent the input channels for the spatial augmentation block. A factor n increases the number of channels. Factor n was used with a value of 4, as it was found that it provided the best result. Finally, a PS was used to rearrange the view image stack of size (64, H, W) into a raw LF image of size (8H, 8W) to be applied to the angular augmentation block to generate the final image LF_HR_.

#### 3.3.2. Loss Function

Our network was trained to reconstruct a high-resolution (HR) LF raw image LF_HR_ given a low-resolution (LR) LF raw image LF_LR_. The learning process can be stated as follows:

$${\mathrm{LF}}_{\mathrm{HR}}=f\left({\mathrm{LF}}_{\mathrm{LR}},\theta \right)$$

The function that reconstructs the HR image from its counterpart LR image is represented by *f*(·), implemented by the proposed network, and θ denotes the network parameters learned during training.

We trained our network to reduce the *L*_1_ loss (the sum of all the absolute differences) between the reconstructed HR image and its corresponding ground-truth image. When a training set contains N combinations of input and ground-truth pictures, the *L*_1_ loss is defined as follows:

$$L_{1}\left(\theta \right)=\frac{1}{N}\overset{N}{\underset{i=1}{\sum }}{\left|{\mathrm{LF}}_{\mathrm{HR}}^{i}-f\left({\mathrm{LF}}_{\mathrm{LR}}^{i}\right)\right|}_{1}$$

#### 3.3.3. Training Details

We trained the proposed model and assessed the results using PSNR and SSIM [40] on the Y channel (luminance) of the converted YCbCr space. Moreover, 3 × 3 2D convolutional kernels were used to build our network with zero-padding and 64 filters in each convolution layer. We used 100 LF images to train our model. These images were captured by Lytro Illum cameras and made publicly available [15,41]. The spatial resolution of the images was 376 × 541, and the angular resolution was 14 × 14. We trained our model on central 8 × 8 views to avoid optical distortion and light falloff. First, we constructed input and corresponding ground-truth patches. We extracted patches (32 × 32 in size) with one stride from each view to obtain input patches of size (4, 32, 32) and output patches of size (256, 256). Our training dataset has 17,600 pairs of input and ground-truth patches, which is adequate for the training. Our model was trained with a batch size of 32 by ADAM optimizer [42] with β1 = 0.9, β2 = 0.999, and ǫ = 10^−8^. The initial learning rate was set to 10^−4^ and dropped exponentially by 0.1 per 100 epochs. We used TensorFlow [43] to train our model for 150 epochs on an NVIDIA GeForce RTX 3090 GPU.

## 4. Experiments and Discussion

### 4.1. Comparison with the State-of-the-Art

The proposed framework is compared to cutting-edge learning-based LF reconstruction approaches, such as Kalantari et al. [15], Shi et al. [44], Yeung et al. [45], and Zhang et al. [17]. Real-world LF datasets, such as 30 scenes (30 LFs) [15], refractive and reflective surfaces (31 LFs), and occlusions (43 LFs) from the Stanford Lytro Light Field Archive were used for the evaluation [41]. The overall reconstructed views’ average PSNR and SSIM [43] of the luminance (Y channel) were calculated to quantify the reconstruction quality. For the evaluation, we used one interpolation task (task 1: 2 × 2 − 8 × 8 extrapolation 0), and two extrapolation tasks (task 2: 2 × 2 − 8 × 8 extrapolation 1, and task 3: 2 × 2 − 8 × 8 extrapolation 2).

#### Interpolation Task (2 × 2 − 8 × 8 Extrapolation 0)

Table 1 summarizes the numerical results in terms of (PSNR/SSIM). The results demonstrate that the proposed model achieves the highest reconstruction quality, with average PSNR gains of 0.35, 1.12, 1.21, and 2.22 dB, with average PSNR increases over Zhang et al. [17], Yeung et al. [45], Shi et al. [44], and Kalantari et al. [15], respectively. Additionally, our model has the best SSIM on two datasets and a very close second-best result on one dataset. Kalantari et al. [15] and Shi et al. [44] create novel views by warping the input views based on their estimated disparity. However, on the other hand, depth estimation and warping are very difficult, especially for LF images with a small difference in depth, making it easy to have flaws and look out of place. Due to Yeung et al.’s [45] disregard for the links between separate views, their technique results in false shadows and ghosting artifacts along the boundaries of reconstructed views. Zhang et al. [17] explored additional LF information using micro-lens images and view image stacks, and their suggested model performs well.

**Figure 5:**
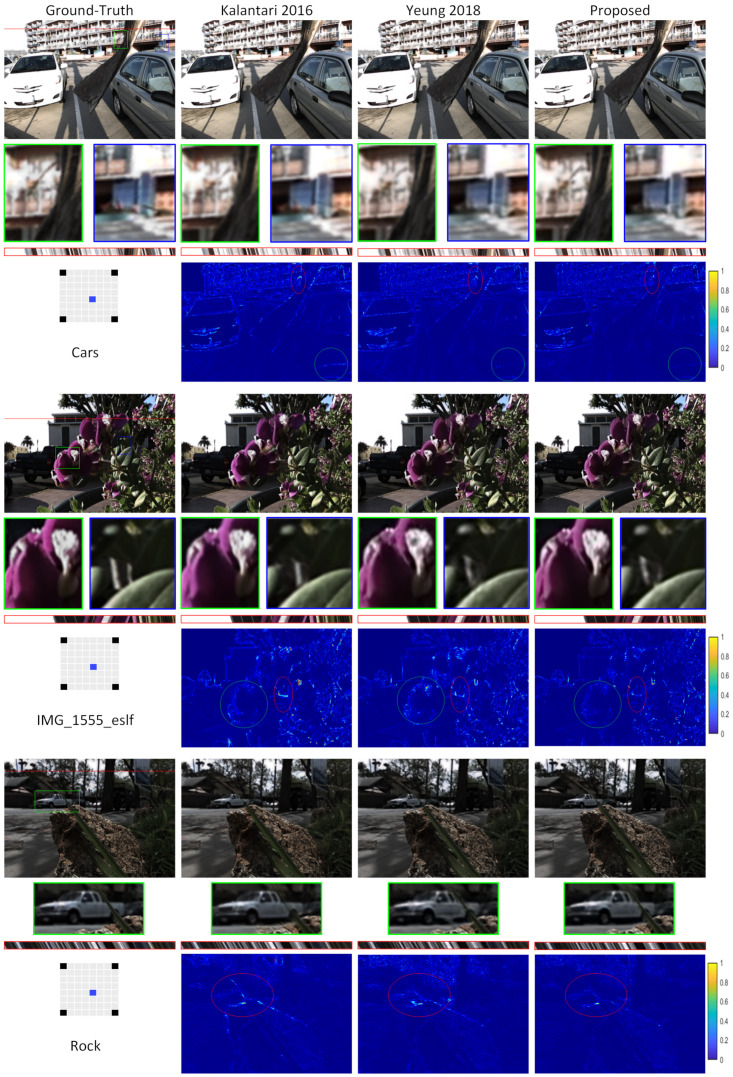
Comparison of LF image reconstruction to other approaches using the relevant ground-truth images on task 1: 2 × 2 − 8 × 8 extrapolation 0 [15,45]. Additionally, error maps are given between the reconstructed LF images and the relevant ground truth images. A diagram shows the input, output, and images to be reconstructed relationships on the left-hand side. Red boxes indicate the extracted EPIs, whereas blue and green boxes indicate a close-up of reconstructed LF image parts. The performance of our strategy is proven through error maps, for example, by the red ellipse around the tree limb in the Cars scene. The red ellipse in the rock scene denotes the complicated area of the automobile with the rock barrier and the longitudinal leaf.

Using raw LF images, the network can interpret and model the relationship between several views of the same scene, recovering more features and providing a better quality image. In addition to that, the use of this proposed residual model eases passing the low-frequency information through skip connections to the final output. The main parts of the network can instead focus and train on extracting more features and restoring high-frequency components. Figure 5 illustrates a visual comparison between our model’s reconstructions and two other models. Even when obscuring objects or the backdrop is complicated, our approach can reconstruct higher-quality images with distinct margins around object boundaries. Error maps provide a more accurate comparison of the reconstructed pictures. For instance, the red ellipse indicates the automobile’s difficult area error with the rock barrier and longitudinal leaf in the rock scene.

### 4.2. Extrapolation Tasks (2: 2 × 2 − 8 × 8 Extrapolation 1, 2)

Reconstructing 8 × 8 out of 2 × 2 views is a challenging task due to the sparseness of the input views. Yeung et al. [45] observed that the reconstruction quality of the center views is much worse than that of the views located near the input views, as shown in Figure 5 in [45]. Because the center view is the farthest distance from any input views, inferring the details with greater accuracy presents the biggest problem. Therefore, they proposed different combinations of interpolation and extrapolation to reconstruct LF images. As a result, the average distance from all the novel views is shorter than before, increasing the reconstruction quality of the center views.

Most available algorithms are optimized for interpolation tasks and cannot predict extrapolated views. That is why ghosting and artifacts often appear around thin structures and occluded regions. Extrapolation is more challenging than interpolation because certain portions of the reconstructed views are not present in the input views. In addition, it cannot keep the slopes of the lines in the reconstructed EPIs the same. It is challenging to devise a method for dealing with different relationships between input and output views. However, with our proposed approach using the raw LF image, the task becomes more feasible and efficient. The numerical results for the extrapolation tasks are summarized in Table 2 and Table 3. The results reveal that the proposed model outperforms state-of-the-art approaches in terms of PSNR and SSIM.

### 4.3. Ablation Study

In the case of the first reconstruction task (extrapolation 0), we evaluated four different designs to demonstrate the influence of the model’s various components, as indicated in Table 4. First, we started with a model with the LFR block only, and then we compared two different models with the LFR block and one type of augmentation block only. Finally, we checked the proposed model with LFR and LFA blocks. It is clear from the results that the LFR block cannot reconstruct high-quality LFs; however, it can provide good initial LFs. The results demonstrate that although the LFR block is incapable of reconstructing high-quality LFs, it may give adequate initial LFs. When comparing the effect of angular augmentation to spatial augmentation, we find that angular augmentation on the raw LFs gives better results. Still, it is not sufficient to achieve the best performance.

In the case of the second and third reconstruction tasks (extrapolation 1, 2), we evaluated four different designs, as indicated in Table 5 and Table 6. First, we start with a model with the LFR block only, and then we compare by adding one LFA block, two LFA blocks, and three LFA blocks, where the LFA type used here is the angular augmentation. The LFR block is not enough to produce satisfactory results. By adding more augmentation blocks, the model starts fitting. Therefore, we used the LFR block with one LFA block only.

## 5. Limitations and Future Work

We proposed a method to reconstruct LF images using raw LF representation. However, our method works well for some reconstruction tasks, but it cannot be applied to other tasks. For example, we can reconstruct 8 × 8 out of 2 × 2 views in three different ways (extrapolation 0, 1, and 2), but other tasks cannot be applied, such as reconstructing 7 × 7 out of 2 × 2 or out of 3 × 3 views. We want to develop a reconstruction approach that works well for all reconstruction tasks in the future. Despite the improvement brought about by the proposed method, it is marginal and, hence, we will attempt to design another method that produces more accurate results. Additionally, we will attempt to develop a model capable of working with both broad and small baseline LF images since the model suggested in this study is only capable of working with small baseline images.

## 6. Conclusions

We presented a learning-based technique for LF reconstruction. To investigate the non-local properties of 4D LF effectively, we used raw LF representation, which allowed the network to comprehend and model the relationship accurately, hence recovering more texture information and improving quality. Additionally, we presented a residual architecture for problems involving interpolation and extrapolation. We trained our network intending to minimize the *L*_1_ loss between the reconstructed and ground truth images. Experiments on three real-world datasets demonstrate that our proposed model outperforms the state-of-the-art methods.

## Figures and Tables

**Table 1:** Numerical comparison (PSNR/SSIM) of the proposed model for LF reconstruction using the dataset 30 scenes, reflective, and occlusions on task 1: 2 × 2 − 8 × 8 extrapolation 0.

Dataset	Kalantari [15]	Shi [44]	Yeung [45]	Zhang [17]	Proposed
30 Scenes	40.11/0.979	41.12/0.985	41.21/0.982	41.98/0.986	42.33/0.985
Reflective	37.35/0.954	38.10/0.958	38.09/0.959	38.71/0.962	38.86/0.962
Occlusions	33.21/0.911	34.41/0.929	34.50/0.921	34.76/0.918	34.69/0.922
Average	36.89/0.948	37.88/0.957	37.93/0.954	38.48/0.955	38.62/0.956

**Table 2:** Numerical comparison (PSNR/SSIM) of the proposed model for LF reconstruction using the dataset 30 scenes, reflective, and occlusions on task 2: 2 × 2 − 8 × 8 extrapolation 1.

Dataset	Yeung [45]	Zhang [17]	Proposed
30 Scenes	42.47/0.985	43.57/0.989	43.76/0.988
Reflective	41.61/0.973	42.33/0.975	42.44/0.974
Occlusions	37.28/0.934	37.61/0.937	37.93/0.948
Average	40.45/0.964	41.17/0.967	41.38/0.970

**Table 3:** Numerical comparison (PSNR/SSIM) of the proposed model for LF reconstruction using the dataset 30 scenes, reflective, and occlusions on task 3: 2 × 2 − 8 × 8 extrapolation 2.

Dataset	Yeung [45]	Zhang [17]	Proposed
30 Scenes	42.74/0.986	43.41/0.989	43.43/0.987
Reflective	41.52/0.972	42.09/0.975	42.26/0.975
Occlusions	36.96/0.937	37.60/0.944	37.91/0.945
Average	40.41/0.965	41.03/0.969	41.20/0.969

**Table 4:** In investigating the proposed architecture, including the spatial and angular augmentation blocks, where ✓ means this augmentation block while X not using it. We observe the best PSNR (dB) and SSIM values on three real-world LF datasets.

Spatial	Angular	30 Scenes	Reflective	Occlusions	Average
X	X	36.69/0.958	36.02/0.944	31.35/0.888	34.69/0.930
✓	X	37.30/0.963	36.36/0.946	31.60/0.891	35.09/0.933
X	✓	41.53/0.981	38.62/0.959	34.46/0.926	38.20/0.955
✓	✓	42.33/0.985	38.86/0.962	34.69/0.922	38.62/0.956

**Table 5:** In investigating the proposed architecture, including the angular augmentation block, we observe the best PSNR (dB) and SSIM values on task 2: 2 × 2 − 8 × 8 extrapolation 1.

Dataset	Inter	Aug1	Aug2	Aug3
30 Scenes	38.72/0.973	43.76/0.988	43.82/0.988	43.88/0.988
Reflective	40.13/0.967	42.44/0.974	42.45/0.975	42.46/0.975
Occlusions	34.50/0.927	37.93/0.948	37.91/0.947	37.98/0.948
Average	37.78/0.955	41.38/0.970	41.39/0.970	41.44/0.970

**Table 6:** In investigating the proposed architecture, including the angular augmentation block, we observe the best PSNR (dB) and SSIM values on task 2: 2 × 2 − 8 × 8 extrapolation 2.

Dataset	Inter	Aug1	Aug2	Aug3
30 Scenes	40.10/0.979	43.43/0.987	43.57/0.987	43.61/0.987
Reflective	39.52/0.967	42.26/0.975	42.29/0.975	42.29/0.975
Occlusions	35.01/0.930	37.91/0.945	38.01/0.946	37.93/0.945
Average	38.21/0.959	41.20/0.969	41.29/0.969	41.28/0.969

## Data Availability

The datasets used in this paper are public datasets. We also provide the test and the evaluation codes of the proposed method at: https://github.com/ahmeddiefy/E2E_LF_RAW, which was created (accessed on 8 April 2022).
